# Long‐term follow‐up of chronic central serous chorioretinopathy patients receiving oral eplerenone and half‐dose photodynamic therapy in the SPECTRA trial: SPECTRA trial report No. 4

**DOI:** 10.1111/aos.70106

**Published:** 2026-02-11

**Authors:** Helena M. A. Feenstra, Issam El Mansori, Thomas J. van Rijssen, Roula Tsonaka, Roselie M. H. Diederen, Carel B. Hoyng, Reinier O. Schlingemann, Camiel J. F. Boon, Elon H. C. van Dijk

**Affiliations:** ^1^ Department of Ophthalmology Leiden University Medical Center Leiden the Netherlands; ^2^ Department of Biomedical Data Sciences Leiden University Medical Center Leiden the Netherlands; ^3^ Department of Ophthalmology Amsterdam University Medical Center, University of Amsterdam Amsterdam the Netherlands; ^4^ Department of Ophthalmology Radboud University Medical Center Nijmegen the Netherlands; ^5^ Department of Ophthalmology Fondation Asile des Aveugles, Jules‐Gonin Eye Hospital, University of Lausanne Lausanne Switzerland; ^6^ Department of Vitreoretinal Surgery Rotterdam Eye Hospital Rotterdam the Netherlands

**Keywords:** central serous chorioretinopathy, eplerenone, half‐dose photodynamic therapy, randomized controlled trial, subretinal fluid

## Abstract

**Purpose:**

Assessing the 24‐month treatment outcome of half‐dose photodynamic therapy (PDT) or oral eplerenone in chronic central serous chorioretinopathy (cCSC).

**Methods:**

Multicentre randomized clinical trial included cCSC patients from the SPECTRA trial who were randomized to receive half‐dose PDT or oral eplerenone. Outcomes were evaluated at 24 months, including the primary outcome measure of complete resolution of subretinal fluid (SRF) on optical coherence tomography (OCT) and the secondary outcome measures best‐corrected visual acuity (BCVA), retinal and foveal sensitivity on microperimetry and the National Eye Institute Visual Functioning Questionnaire 25 (NEI‐VFQ25) score.

**Results:**

At baseline, 107 patients were included, of which 80 patients attended the final visit at 24 months: 40 had been randomized to primary treatment with half‐dose PDT and 40 to eplerenone. In the eplerenone group, as many as 36/40 (90%) needed crossover treatment at 3 months with half‐dose PDT due to persistent SRF, compared to 8/40 (20%) in the half‐dose PDT group. At the final visit, complete SRF resolution was observed in 32/40 (80%) who were primarily treated with half‐dose PDT and in 35/40 (88%) who received primary eplerenone treatment (*p* = 0.609). There were no significant differences in terms of BCVA, foveal sensitivity, retinal sensitivity and NEI‐VFQ25 score.

**Conclusions:**

This study provides evidence for the long‐term efficacy of half‐dose PDT in resolving SRF and improving functional outcomes in cCSC. Even when patients initially received eplerenone treatment that did not result in complete SRF resolution, crossover treatment with half‐dose PDT led to similar (long‐term) group outcomes compared to half‐dose PDT as initial treatment.

## 1 INTRODUCTION

Central serous chorioretinopathy (CSC) is a relatively common cause of vision loss due to fluid accumulation in the macula, with an estimated incidence of 9.9 per 100.000 in men and 1.7 per 100.000 in woman, although experience from clinical practice indicates higher incidence rates (Feenstra et al., 2024.) CSC can lead to significant visual impairment, including blurred vision, metamorphopsia, micropsia and abnormal colour discrimination, and is associated with a decreased vision‐related quality of life especially in chronic cases (Breukink et al., 2017; Feenstra et al., 2024).

Despite the fact that the pathogenesis of CSC is not yet fully understood, choroidal abnormalities have been thought to be a key factor (Cardillo Piccolino et al., 1995; Daruich et al., 2015; Guyer et al., 1994; Spaide et al., 1996; van Dijk et al., 2020). These choroidal abnormalities include a pachychoroid (a thickened choroid), in combination with pachyvessels (i.e. dilated choroidal vessels in Haller's layer) due to venous overload choroidopathy, and increased choroidal permeability leading to fluid leakage, which can be visualized using multimodal imaging techniques including optical coherence tomography (OCT), fluorescein angiography (FA) and indocyanine green angiography (ICGA) (Cheung et al., 2024; Feenstra et al., 2024; Pauleikhoff et al., 2024a, 2024b; Spaide et al., 2022). There may also be a potential role for choroidal arteriovenous anastomoses (Brinks et al., 2022). It is believed that the aforementioned choroidal abnormalities are responsible for damage to the retinal pigment epithelium (RPE) (Daruich et al., 2015; Spaide et al., 2022). More recently, it has been found that scleral abnormalities might also have a role in CSC occurrence (Imanaga et al., 2021, 2023). The most important risk factors for CSC include male sex, genetic risk variants and use of corticosteroids (de Jong et al., 2015; Kaye et al., 2020; Mohabati, Boon, & Yzer, 2020; Mohabati, Hoyng, et al., 2020; Mohabati, Schellevis, et al., 2018, 2020; Mohabati, van Dijk, et al., 2018; Mohabati, van Rijssen, et al., 2018; van Dijk et al., 2017).

There are two primary types of CSC, which can be distinguished based on the duration of disease and the abnormalities observed on multimodal imaging (Feenstra, van Dijk, et al., 2023; Feenstra, van Dijk, Rijssen, Tsonaka, Diederen, Schlingemann, & Boon, 2022; van Rijssen et al., 2022). However, there is some variability in the reported definitions of these two subtypes, and alternative classifications have been recently proposed, which need more validation (Chhablani et al., 2020, 2022). Acute CSC (aCSC) is often defined by a focal leak on FA (if performed) with very limited atrophic RPE abnormalities. The subretinal fluid (SRF) in aCSC usually resolves spontaneously within a few months without the need for treatment, although the condition may recur in up to 24% of untreated cases (Mohabati, Boon, & Yzer, 2020; Mohabati, Hoyng, et al., 2020; Mohabati, Schellevis, et al., 2020). Conversely, cCSC is characterized by a persistent serous neuroretinal detachment, which can be either small in size or extensive, as well as multifocal in the case of multiple leakage areas. Chronic CSC typically presents with atrophic RPE changes on FA that can range from a single localized area to extensively diffuse atrophic alterations (Mohabati, Schellevis, et al., 2018; Mohabati, van Dijk, et al., 2018; Mohabati, van Rijssen, et al., 2018). In contrast to aCSC, SRF on OCT typically persists for longer than 3–4 months, and one or more focal leakage points are visible on FA (Breukink et al., 2017; Feenstra et al., 2024).

Several large investigator‐initiated randomized clinical trials (RCTs) on the treatment of cCSC have been conducted in recent years, assessing the role of half‐dose photodynamic therapy (PDT), the oral mineralocorticoid receptor antagonist eplerenone and high‐density subthreshold micropulse laser treatment (HSML) (Feenstra et al., 2024). The PLACE trial, which was the first large RCT in cCSC, demonstrated that half‐dose PDT is superior to 810 nm HSML in achieving a complete SRF resolution on OCT, as well as functional improvement (van Dijk et al., 2018). The VICI trial demonstrated that oral eplerenone monotherapy is not superior to placebo in terms of SRF resolution, best‐corrected visual acuity (BCVA) and retinal morphology at 1 year after the baseline visit (Lotery et al., 2020). Subsequently, the SPECTRA trial, which compared half‐dose PDT to oral eplerenone at 3 months after (initiation of) treatment, showed that half‐dose PDT is more effective than eplerenone monotherapy in achieving complete SRF resolution as well as functional improvement in patients with cCSC (van Rijssen et al., 2022). In the subsequent SPECS trial, which included patients from the SPECTRA trial who had persistent SRF after 3 months of primary treatment (with either eplerenone monotherapy or half‐dose PDT), it was found that cCSC patients who received crossover treatment with half‐dose PDT after primary eplerenone treatment had significantly better outcomes in terms of complete SRF resolution on OCT and foveal sensitivity on microperimetry at 6 months after crossover to half‐dose PDT compared to patients who received crossover treatment with eplerenone treatment after unsuccessful initial treatment with half‐dose PDT (Feenstra, van Dijk, et al., 2023; Feenstra, van Dijk, Rijssen, Tsonaka, Diederen, Schlingemann, & Boon, 2022). At 12 months, there was no significant difference in terms of complete SRF resolution between the patients who were primarily randomized to half‐dose PDT and to oral eplerenone. However, it is important to note that 89.6% of cCSC patients who underwent half‐dose PDT achieved complete SRF resolution after 12 months, including those who underwent crossover treatment after unsuccessful eplerenone therapy (Feenstra, Diederen, et al., 2023; Feenstra, van Dijk, et al., 2023). Based on the outcome of these trials, PDT with reduced settings has been found to be the treatment of choice in cCSC, as stated in a recently published evidence‐based treatment guideline for CSC (Feenstra et al., 2024). Still, additional studies with a longer duration of follow‐up are required to assess the longer term outcome of treatment.

The current study presents a follow‐up on the SPECTRA and SPECS trials, reporting the outcome of cCSC patients at 24 months after the first treatment (either eplerenone monotherapy or half‐dose PDT, or both in the case of crossover after unsuccessful initial treatment with half‐dose PDT or oral eplerenone).

## 2 MATERIALS AND METHODS

The current study is a part of a prospective multicentre study, the SPECTRA trial, which is identified by the clinicaltrial.gov identifier NCT03079141. The trial protocol as well as the results after 3 months, after crossover treatment and after 12 months have previously been published. Patients were recruited from three academic tertiary referral centres located in the Netherlands: Leiden University Medical Center (Leiden), Amsterdam University Medical Center (Amsterdam) and Radboud University Medical Center (Nijmegen) (Feenstra, van Dijk, et al., 2023; Feenstra, van Dijk, Rijssen, Tsonaka, Diederen, Schlingemann, & Boon, 2022; van Rijssen et al., 2022). The study followed the principles of the Declaration of Helsinki, and all participants provided written informed consent. The Institutional Review Board Committee approved the study at all participating centres prior to its commencement. Ethics approval for the study was granted by the medical ethical committee of Leiden University Medical Center (NL59158.058.16).

In the SPECTRA trial, cCSC patients aged 18 years or older, who presented with SRF on OCT and typical findings of cCSC on multimodal imaging including FA and ICGA, were randomized into two treatment groups at a 1:1 ratio: oral eplerenone or half‐dose PDT (see Figure S1). The presence of SRF was assessed using OCT at 3 months after treatment initiation (evaluation visit 1) to evaluate its effectiveness. Patients with persistent SRF were eligible for crossover treatment and were evaluated at 3 months after crossover treatment (evaluation visit 2) (Feenstra, van Dijk, et al., 2023; Feenstra, van Dijk, Rijssen, Tsonaka, Diederen, Schlingemann, & Boon, 2022). Patients included in the study were followed up after 12 months from baseline visit (evaluation visit 3) (Feenstra, van Dijk, et al., 2023; Feenstra, van Dijk, Rijssen, Tsonaka, Diederen, Schlingemann, & Boon, 2022).

In the current study, cCSC patients underwent an additional follow‐up at 24 months (final visit) after baseline visit: primary endpoint was the presence of SRF on OCT, whereas secondary endpoints included the functional parameters BCVA in Early Treatment of Diabetic Retinopathy Study (ETDRS) letters, foveal and retinal sensitivity in decibels (dB) using microperimetry and the National Eye Institute Visual Function Questionnaire 25 (NEI‐VFQ‐25) score. Retinal sensitivity was measured by using a Macular Integrity Assessment Microperimeter (CenterVue), with an examination that covers a 10° diameter area with 37 test loci distributed in a radial pattern within the macula. The foveal sensitivity was defined as the mean sensitivity of the central 13 spots located in the fovea.

In addition, this study also evaluated subfoveal choroidal thickness (SFCT) and central retinal thickness (CRT) on OCT. SFCT was defined as the distance between the outer border of the hyperreflective line representing the RPE and the choroidoscleral interface measured at the central foveal depression (Kang et al., 2021; Kumar et al., 2019; van Rijssen, Mohabati, et al., 2018; van Rijssen, van Dijk, et al., 2018). CRT was defined as the distance from the inner internal limiting membrane to the inner border of the ellipsoid zone at the central foveal depression, as proposed by van Rijssen et al. (van Rijssen, Mohabati, et al., 2018; van Rijssen, van Dijk, et al., 2018). At final visit, any signs of new outer retinal or RPE atrophy in the foveal area, as evidenced by increasing irregularity or discontinuation of the external limiting membrane or the ellipsoid zone on OCT, or increasing hypo‐autofluorescence on fundus autofluorescence imaging, both within a diameter of 1500 micrometres of the central foveal depression, were documented. Adverse events observed during the study were registered. In cases of new visual complaints between evaluation visit 3 and final visit, patients consulted their treating ophthalmologist at one of the study sites for an evaluation and—if needed—for additional treatment.

### 2.1 Half dose‐PDT protocol

To perform half‐dose PDT in the study eye, patients first received topical 2.5% phenylephrine and 1% tropicamide to dilate the pupil. Next, an intravenous infusion of 3 mg/m^2^ (half‐dose) verteporfin (Visudyne®; Novartis, Basel, Switzerland) was delivered over a period of 10 minutes. The study eye was then anaesthetized with oxybuprocaine 0.4%, and a magnification lens (1.5× magnification) (Volk Optical) for PDT was placed on the eye. The ocular area to be treated was determined by the central reading centre based on the hyperfluorescent areas of leakage seen on ICGA to avoid interobserver variability on the interpretation of the extent of these choroidal abnormalities. The diameter of the laser spot was based on the diameter of the hyperfluorescent area(s) observed on ICGA plus an additional 1 mm. Finally, half‐dose PDT was performed on the determined area with a fluency of 50 J/cm^2^, a wavelength of 689 nm and a treatment duration of 83 seconds (van Dijk et al., 2020; van Rijssen et al., 2022).

### 2.2 Oral eplerenone protocol

The eplerenone treatment protocol involved the oral administration of 25 mg per day (Inspra®, Pfizer, Capelle aan den IJssel, the Netherlands) for 1 week, while concurrently monitoring serum potassium levels. Thereafter, the dosage was adjusted based on the serum potassium levels as follows: increased to 50 mg per day for serum potassium levels less than 5.0 mEq/L, maintained at 25 mg per day for serum potassium levels between 5.0 and 5.4 mEq/L, reduced to 25 mg every 2 days for serum potassium levels between 5.5 and 5.9 mEq/L and terminated if serum potassium levels were equal to or higher than 6.0 mEq/L. At 1, 2 and 3 months after the start of eplerenone treatment, the patient's serum potassium was measured again, during which the dose of eplerenone was adjusted based on the serum potassium level (van Rijssen et al., 2022). Using eplerenone for the treatment of cCSC is off‐label.

### 2.3 Statistical analysis

The statistical analyses for this study were conducted using both SPSS statistics (version 29.0; IBM Corp, Armonk, New York, USA) and R (version 4.0.1; R Foundation for Statistical Computing, Vienna, Austria). The primary endpoint, which was the binary longitudinal outcome of SRF on OCT, was analysed using a mixed effects logistics regression model with the function mixed_model(.) from the R package GLMMadaptive. The continuous longitudinal endpoints, including BCVA, foveal and retinal sensitivity on microperimetry, NEI VFQ‐25 score, CRT and SFCT, were analysed using a linear mixed effects model in SPSS.

NEI VFQ‐25 responses were transformed into a score ranging from 0 to 100 to facilitate statistical analysis. Mixed effects models were used to account for within‐subject correlations resulting from the repeated evaluations in time. Moreover, mixed effects models were used to provide valid results under the Missing At Random assumption for the missing evaluation visits.

It should be noted that patients were not analysed in four groups, taking crossover treatment into account, since crossover treatment was not given at random, which would introduce bias in the analysis of the four separate groups using a mixed model. Therefore, the intention‐to‐treat analysis was performed by analysing patients in the subgroup to which they were originally randomized, irrespective of the crossover treatment received, after consultation of the statistician involved in the current study.

## 3 RESULTS

**FIGURE 1:**
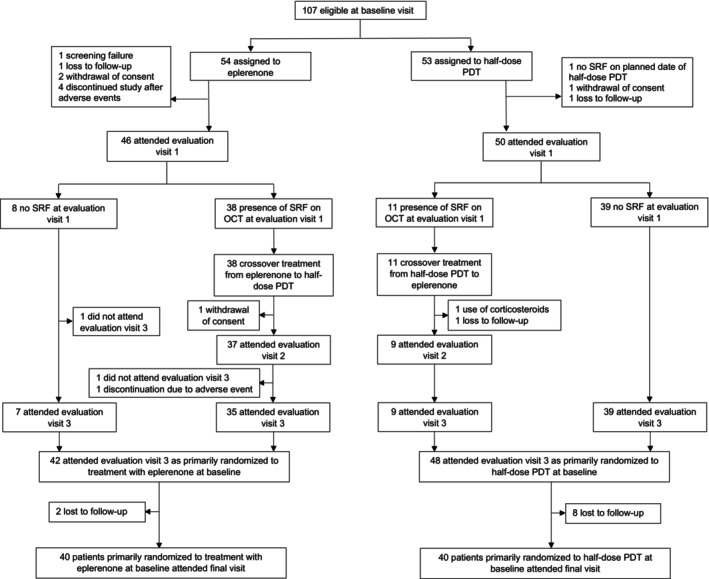
Flow chart depicting the selection criteria for the patients in the SPECTRA trial (half‐dose photodynamic therapy versus eplerenone in chronic central serous chorioretinopathy). OCT, optical coherence tomography; PDT, photodynamic therapy; SRF, subretinal fluid.

In the SPECTRA trial, 107 cCSC patients were evaluated at 3 months after baseline visit (see Figure 1). Among patients who received half‐dose PDT, 22.0% had persistent SRF on OCT at evaluation visit 1, as compared to 82.6% of oral eplerenone patients (van Rijssen et al., 2022). According to the study protocol, crossover treatment was performed in the patients with persistent SRF. Following initial half‐dose PDT, 48 patients were assessed at evaluation visit 3 (at 12 months after baseline), and 42 patients were assessed after initial eplerenone treatment.

For the present study, conducted at 24 months after the baseline visit (final visit), a total of 27 patients could not be included at final visit due to loss to follow‐up, withdrawal of consent, discontinuation after adverse events or screening failure. This resulted in a remaining sample of 80 patients at final visit. Of these 80 patients, 40 patients were initially randomized to eplerenone treatment, while the other 40 were randomized to half‐dose PDT. Of the 40 patients who were originally randomized to eplerenone treatment, 32 (80%) had also received crossover treatment with half‐dose PDT because of persistent SRF at evaluation visit 1. Of the patients who were primarily randomized to half‐dose PDT, 8 (20%) had received crossover treatment with eplerenone. Notably, one patient who missed evaluation visit 3 for unknown reasons but participated in the final visit was still included in the analyses.

At baseline visit, the mean age of the patients who were primarily randomized to eplerenone (47.5 years) was not statistically significantly different from that of the patients randomized to half‐dose PDT (44.5 years). Both groups predominantly consisted of male patients (94%), and there were no significant differences between the groups in terms of mean BCVA, mean retinal sensitivity and mean NEI VFQ‐25 composite score (see Table S1; van Rijssen et al., 2022).

In the current study, it was observed that 80% (32 out of 40) of the patients who were primarily randomized to half‐dose PDT had no SRF on OCT at final visit, which was a significant improvement compared to baseline visit (*p* < 0.001). Likewise, 87.5% (35 out of 40) of the patients who were primarily randomized to receive eplerenone (of whom 32/40 (80%) patients had subsequently received half‐dose PDT as crossover treatment due to persistent SRF on OCT at evaluation visit 1 despite eplerenone treatment) had no SRF on OCT at final visit, which was also a significant improvement compared to baseline visit (*p* < 0.001). There was no difference in the odds ratio for the presence of SRF on OCT at final visit between the patients primarily randomized to half‐dose PDT and those primarily randomized to eplerenone (odds ratio 1.356, 95% CI 0.419–4.407 *p* = 0.609) (see Table 1).

**TABLE 1:** Primary and secondary outcome measures of the SPECTRA trial (half‐dose photodynamic therapy versus eplerenone in chronic central serous chorioretinopathy) at baseline visit, evaluation visit 1 (3 months), evaluation visit 3 (12 months) and final visit (24 months).

Variable	Visit	Primarily randomized to half‐dose PDT*	Primarily randomized to eplerenone*	*p*‐Value**
Absence of SRF on OCT	Evaluation visit 1	39/50 (78.0%)	8/46 (17.4%)	<0.001
	Evaluation visit 3	43/48 (89.6%)	37/42 (88.1%)	0.522
	Final visit	32/40 (80.0%; *p* < 0.001)	35/40 (87.5%; *p* < 0.001)	0.609
Odds ratio of presence of SRF on OCT at 24 months compared to baseline	Final visit	0.00325 (*p* < 0.001)	0.00239 (*p* < 0.001)	0.609
BCVA (ETDRS letters)	Baseline visit	78.0 (*n* = 53; SD = 13.1)	80.5 (*n* = 54; SD = 7.9)	
	Evaluation visit 1	83.7 (*n* = 50; SD = 10.8; *p* < 0.001)	82.8 (*n* = 46; SD = 9.0; *p* = 0.017)	0.584
	Evaluation visit 3	85.6 (*n* = 48; SD = 11.5; *p* < 0.001)	83.7 (*n* = 42; SD = 11.0, *p* = 0.019)	0.445
	Final visit	83.9 (*n* = 40; SD = 13.7; *p* < 0.001) *p* = 0.414***	84.6 (*n* = 40; SD = 11.7; *p* = 0.007) *p* = 0.460***	0.823
Foveal sensitivity on microperimetry (dB)	Baseline visit	20.3 (*n* = 49; SD = 4.6)	20.0 (*n* = 50; SD = 4.7)	
	Evaluation visit 1	24.2 (*n* = 47; SD = 5.5; *p* < 0.001)	21.7 (*n* = 44; SD = 4.9; *p* = 0.042)	0.023
	Evaluation visit 3	25.5 (*n* = 46; SD = 5.5; *p* < 0.001)	25.0 (*n* = 41; SD = 5.0; *p* < 0.001)	0.380
	Final visit	26.5 (*n* = 40; SD = 3.9; *p* < 0.001) *p* = 0.142***	25.6 (*n* = 40; SD = 5.3; *p* < 0.001) *p* = 0.059***	0.439
Retinal sensitivity on microperimetry (dB)	Baseline visit	22.7 (*n* = 49; SD = 4.3)	22.5 (*n* = 50; SD = 4.1)	
	Evaluation visit 1	25.4 (*n* = 48; SD = 3.4; *p* < 0.001)	23.8 (*n* = 44; SD = 4.0; *p* = 0.044)	0.056
	Evaluation visit 3	26.8 (*n* = 46; SD = 4.0 *p* < 0.001)	25.9 (*n* = 41; SD = 3.7; *p* < 0.001)	0.157
	Final visit	27.3 (*n* = 40; SD = 2.9; *p* < 0.001) *p* = 0.318***	26.0 (*n* = 40; SD = 3.8; *p* < 0.001) *p* = 0.369***	0.091
NEI VFQ‐25 score (points)	Baseline visit	81.7 (*n* = 53; SD = 11.3)	79.5 (*n* = 54; SD = 13.1)	
	Evaluation visit 1	87.2 (*n* = 50; SD = 8.5; *p* < 0.001)	83.8 (*n* = 46; SD = 12.1; *p* < 0.001)	0.096
	Evaluation visit 3	88.7 (*n* = 48; SD = 9.1; *p* < 0.001)	87.8 (*n* = 42; SD = 9.8; *p* < 0.001)	0.534
	Final visit	91.0 (*n* = 40; SD = 8.6; *p* < 0.001) *p* = 0.102***	89.2 (*n* = 40; SD = 10.8; *p* < 0.001) *p* = 0.181***	0.549
Subfoveal choroidal thickness affected eye (μm)	Baseline visit	363.9 (*n* = 34; SD = 77.1)	403.2 (*n* = 37; SD = 128.8)	
	Evaluation visit 1	320.2 (*n* = 31; SD = 76.8; *p* < 0.001)	397.3 (*n* = 31; SD = 116.0; *p* = 0.767)	<0.001
	Evaluation visit 3	342.1 (*n* = 35; SD = 76.3; *p* = 0.073)	359.2 (*n* = 34; SD = 100.2; *p* = 0.020)	0.224
	Final visit	330.3 (*n* = 32; SD = 73.6; *p* = 0.029) *p* = 0.246***	351.6 (*n* = 30; SD = 111.7; *p* = 0.042) *p* = 0.896***	0.108
Central retinal thickness affected eye (μm)	Baseline visit	107.6 (*n* = 53; SD = 20.0)	104.0 (*n* = 54; SD = 19.0)	
	Evaluation visit 1	115.1 (*n* = 50; SD = 25.1; *p* = 0.034)	114.0 (*n* = 53; SD = 25.2; *p* < 0.001)	0.646
	Evaluation visit 3	119.0 (*n* = 48; SD = 25.5; *p* = 0.001)	117.9 (*n* = 42; SD = 24.8; *p* < 0.001)	0.875
	Final visit	122.1 (*n* = 39; SD = 25.0; *p* = 0.002) *p* = 0.204***	121.1 (*n* = 39; SD = 27.0; *p* < 0.001) *p* = 0.126***	0.950

Abbreviations: BCVA, best‐corrected visual acuity; dB, decibel; ETDRS, Early Treatment of Diabetic Retinopathy Study; NEI VFQ‐25, National Eye Institute Visual Functioning Questionnaire 25; OCT, optical coherence tomography; PDT, photodynamic therapy; SD, standard deviation; SRF, subretinal fluid.

* *p*‐Value are compared to the baseline of the treatment group.

** *p*‐Value comparing differences between baseline and final visit between the two treatment groups.

*** *p*‐Value comparing differences between evaluation visit 3 and final visit.

Of the 13 patients who had SRF at final follow‐up, seven did not have SRF at evaluation visit 3 (at 12 months). This group comprised two patients only treated with primary treatment with half‐dose PDT, one patient only treated with primary treatment with oral eplerenone, two patients who received crossover treatment with eplerenone and, lastly, two patients who received crossover treatment with half‐dose PDT. Four patients did have SRF at both evaluation visit 3 and final visit. Two patients did not attend evaluation visit 3, but did have persistent SRF after crossover treatment (evaluation visit 2).

The BCVA at final visit in the group primarily randomized to half‐dose PDT was 83.9 ETDRS letters, which was a significant improvement of +5.9 letters compared to baseline visit (*p* < 0.001). The BCVA at final visit in the group primarily randomized to eplerenone was 84.6 ETDRS letters, showing a significant improvement of +4.1 letters at final visit compared to baseline visit (*p* = 0.007). There was no significant difference in improvement of BCVA between the two groups at final visit (*p* = 0.823).

Both groups showed a significant increase in foveal sensitivity on microperimetry compared to the baseline visit, with an increase of +6.2 dB and +5.6 dB in the patients primarily randomized to half‐dose PDT and eplerenone, respectively (*p* < 0.001 for both groups). Although the group primarily randomized to half‐dose PDT had a slightly higher foveal sensitivity compared to the patients primarily randomized to eplerenone at final visit (26.5 dB vs 25.6 dB), there was no statistically significant difference between the two groups (*p* = 0.439).

Both groups exhibited a significant increase in retinal sensitivity at final visit, when compared to their baseline visit measurements, with the initial half‐dose PDT and eplerenone groups demonstrating an increase of +4.6 and +3.5 dB, respectively (*p* < 0.001 in both groups), a difference that was not statistically significant between the groups (*p* = 0.091).

The group primarily randomized to half‐dose PDT and the group primarily randomized to eplerenone showed an increase in NEI VFQ‐25 score of +9.3 and +9.7 points, respectively, at final visit compared to baseline visit (both *p* < 0.001), although this difference between the groups was not significant (*p* = 0.549).

**FIGURE 2:**
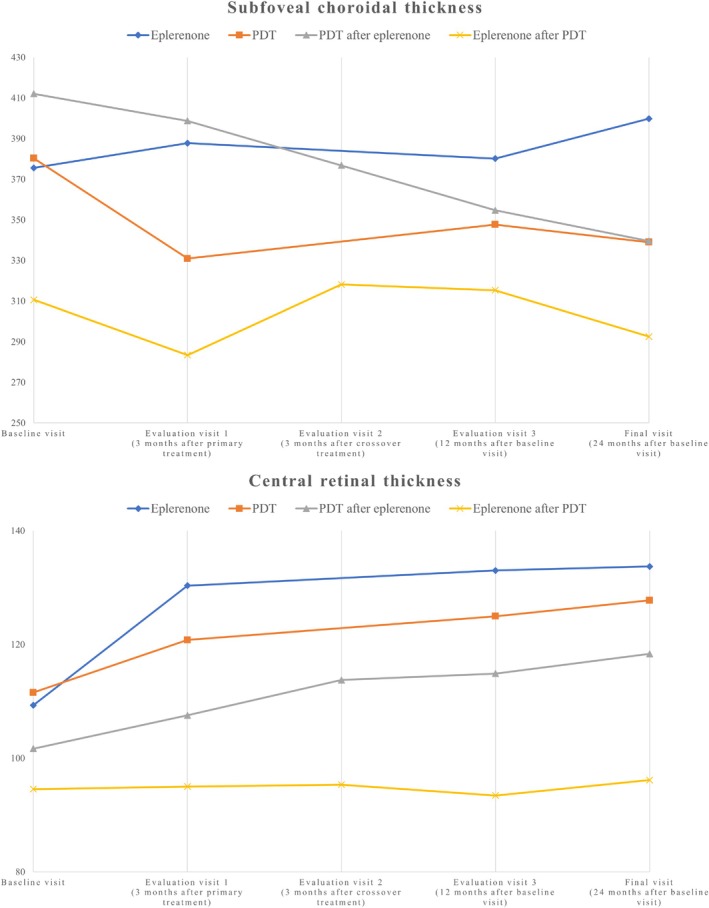
Line graphs of central retinal thickness (CRT) and subfoveal choroidal thickness (SFCT) in different treatment groups of the SPECTRA trial (half‐dose photodynamic therapy versus eplerenone in chronic central serous chorioretinopathy). Three months after primary treatment, a larger decrease in SFCT is seen in the cCSC patient groups who were treated with half‐dose photodynamic therapy (PDT) compared to eplerenone. At 3 months after possible crossover treatment, at evaluation visit 2, an increase in SFCT is seen in patients who received crossover treatment with eplerenone whereas patients who received crossover treatment with half‐dose PDT showed a tendency of a further decline in SFCT. At final visit, a decrease in SFCT was observed in all groups, except for the patients who solely received eplerenone treatment. There were no significant differences in CRT between the groups at any visit.

Patients who were primarily randomized to half‐dose PDT exhibited a significant reduction in SFCT from baseline visit compared to evaluation visit 1 (after solely primary treatment), but no significant reduction in SFCT was seen in the eplerenone group, with a mean decrease of SFCT of −43.5 and −5.9 μm, respectively (*p* < 0.001 and *p* = 0.767) (see Figure 2 and Table 1). The difference in reduction of SFCT at evaluation visit 1 between these two groups was statistically significant (*p* < 0.001). At evaluation visit 3, after 36 of 40 eplerenone patients had received the crossover therapy, a significant reduction in SFCT of −44.0 μm was observed for the first time (*p* = 0.020), and at final visit, there was still a significant reduction of −51.6 μm compared to baseline visit in this patient group who initially received eplerenone (*p* = 0.042). There were no significant differences in reduction of SFCT between the two groups at final visit (*p* = 0.108).

Similarly, an increase in CRT was observed in both the half‐dose PDT group and the eplerenone group at final visit compared to baseline visit, with an increase of +14.5 and +17.1 μm, respectively (see Figure 2). The increase in the half‐dose PDT group was statistically significant compared to baseline visit (*p* = 0.002), as was the increase in the eplerenone group (*p* < 0.001), with no statistically significant difference between the two groups at final visit (*p* = 0.950).

There were no statistical differences between evaluation visit 3 and final visit in any of the secondary parameters (BCVA, foveal sensitivity, retinal sensitivity, NEI VFQ‐25 score, SFCT and CRT) within both groups.

We have previously described the adverse events that occurred between the baseline visit and evaluation visit 1, with 8/54 (15%) patients discontinuing treatment due to adverse events that occurred shortly after the start of the eplerenone treatment, as well as between evaluation visit 1 and evaluation visit 3 (Feenstra, van Dijk, et al., 2023; Feenstra, van Dijk, Rijssen, Tsonaka, Diederen, Schlingemann, & Boon, 2022; van Rijssen et al., 2022; van Rijssen, Mohabati, et al., 2018; van Rijssen, van Dijk, et al., 2018). In the group of patients randomized to half‐dose PDT, 16 of 53 patients (30%) experienced an adverse event between baseline and final visit. None of these events were related to the treatment. Similarly, in the eplerenone group, 30 of 54 patients (56%) had an adverse event throughout the trial, but these events were also unrelated to the treatment (see Table S2). During the course of this study, a total of eight patients (7.5%) developed a macular neovascularization (MNV) in the study eye, three in the group of patients randomized to half‐dose PDT and five in the group randomized to eplerenone. Importantly, all eight patients who developed an MNV had received crossover treatment due to persistent SRF at evaluation visit 1 at 3 months. None of the patients showed an increase in foveal atrophy at the final visit.

## 4 DISCUSSION

The current study is an extension of the SPECTRA and SPECS trials, of which the outcome of (crossover treatment with) eplerenone and half‐dose PDT in cCSC patients at 3 and 12 months has been published before (Feenstra, van Dijk, et al., 2023; Feenstra, van Dijk, Rijssen, Tsonaka, Diederen, Schlingemann, & Boon, 2022; van Rijssen et al., 2022). An important question addressed in the current study is whether there is a difference in complete SRF resolution between patients who received primary eplerenone treatment and those who received primary half‐dose PDT, at 24 months after (start of) primary treatment. Additionally, the study investigated whether a 3‐month delay before initiating crossover therapy (i.e. half‐dose PDT after unsuccessful eplerenone treatment, which was necessary in 38/46 (83%) of patients randomized to primary eplerenone treatment) had any impact on the resolution of SRF and functional endpoints on long‐term follow‐up.

**FIGURE 3:**
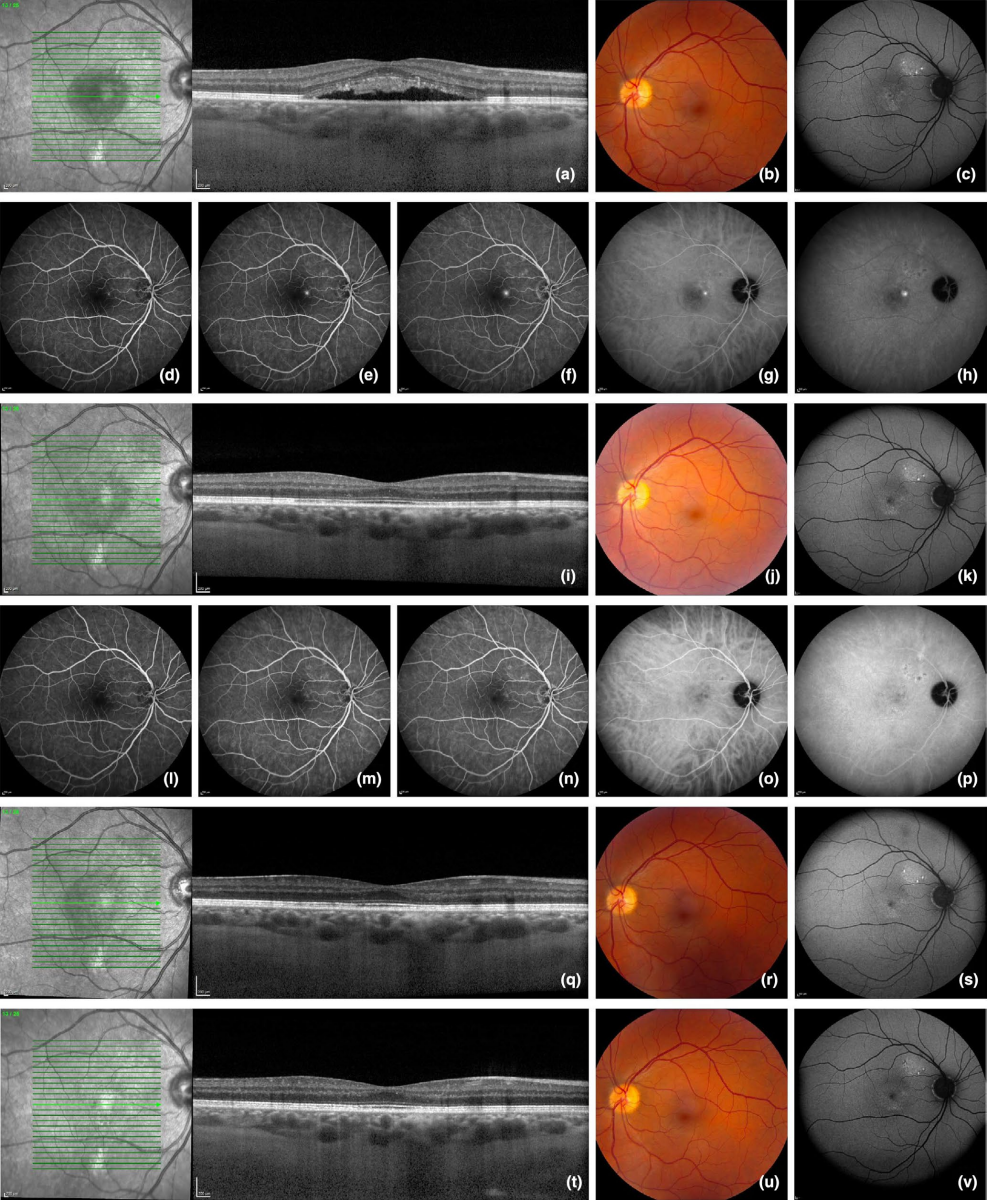
Multimodal imaging of a 38‐year‐old man with chronic central serous chorioretinopathy who received primary half‐dose photodynamic therapy (PDT) without additional crossover treatment in the SPECTRA trial (half‐dose photodynamic therapy versus eplerenone in chronic central serous chorioretinopathy). At the baseline visit of the SPECTRA trial, subretinal fluid (SRF) was observed on optical coherence tomography (OCT; a) and fundus photograph (b). On fundus autofluorescence imaging (FAF; c), both hyper‐ and hypo‐autofluorescent changes were visible. An area of focal leakage increasing in intensity over time was seen on fluorescein angiography (FA; d–f; early, mid‐ and late‐phase, respectively). Indocyanine green angiography (ICGA) showed areas of hyperfluorescent abnormalities with indistinct borders characteristic of central serous chorioretinopathy (g and h, mid‐ and late‐phase, respectively). At 3 months after primary treatment with half‐dose PDT, complete SRF resolution was observed on OCT (i), which was also visible on the fundus photograph (j). No large differences were seen on FAF at 3 months (k). On FA, no leakage of fluorescein was seen anymore (l‐n; early‐, mid‐ and late‐phase, respectively), whereas ICGA showed hypo‐ and hyperfluorescent changes (o and p). At 12 and 24 months after baseline visit (at evaluation visit 3 and final visit, q‐s and t‐v, respectively), SRF on OCT was still absent (q and t). No marked changes were seen on fundus photograph (r and u) and FAF (s and v), compared to the first evaluation visit after half‐dose PDT.

**FIGURE 4:**
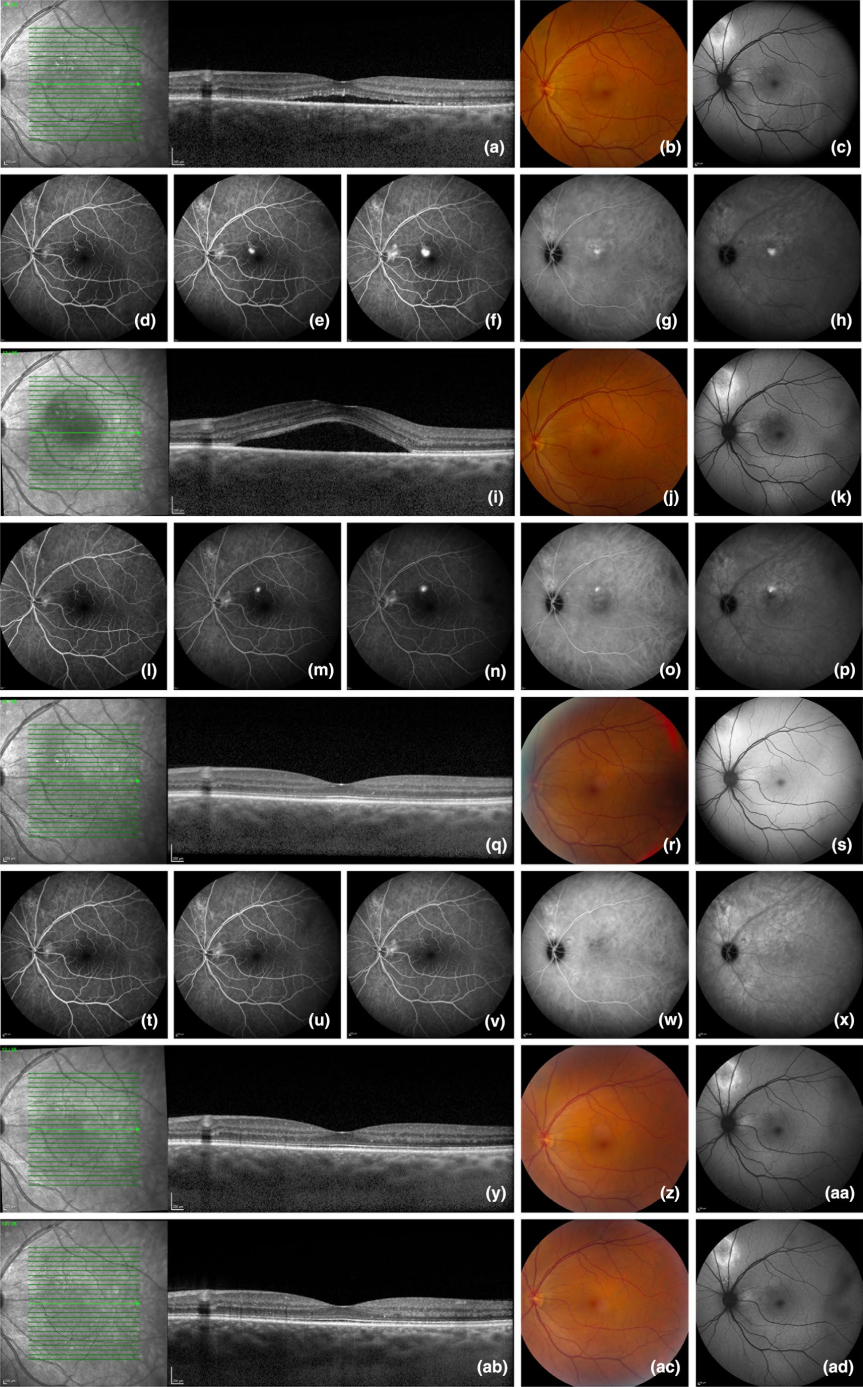
Multimodal imaging of a 53‐year‐old man with chronic central serous chorioretinopathy who received primary treatment with eplerenone and crossover treatment with half‐dose photodynamic therapy (PDT) in the SPECTRA trial (half‐dose photodynamic therapy versus eplerenone in chronic central serous chorioretinopathy). Patient presented with subretinal fluid (SRF) on optical coherence tomography (OCT; a) at the baseline visit of the trial, which was also visible on the fundus photograph (b). Fundus autofluorescence (FAF; c) showed hyper‐ and hypo‐autofluorescent changes. Two focal leakage points were visible on fluorescein angiography (FA), increasing in intensity over time (d‐f, early, mid‐ and late‐phase, respectively). Hyper‐ and hypofluorescent changes were seen on indocyanine‐green angiography (ICGA), with the indistinct border that is characteristic of diseases that are part of the pachychoroid spectrum (g and h, mid‐ and late‐phase, respectively). Three months after the initiation of treatment with eplerenone, SRF was still visible on OCT (i) and fundus photography (j). On FAF (k), hypo‐ and hyperautofluorescent changes were still present. The picture on FA had not markedly changed then (l‐n; early‐, mid‐ and late‐phase, respectively). Moreover, hyper‐ and hypofluorescent changes were still seen on ICGA (o and p, mid‐ and late‐phase, respectively). This patient received crossover treatment with half‐dose PDT, after which complete resolution of SRF had occurred on OCT (q) and the fundus photograph (r) at 3 months after crossover treatment. No large differences were observed in hyper‐ and hypo‐autofluorescence on FAF (s). At that point, no leakage points on FA were visible anymore (t‐v; early‐, mid‐ and late‐phase, respectively). On ICGA, hyper‐ and hypofluorescence was still observed, although the central hyperfluorescence had decreased (w and x, mid‐ and late‐phase, respectively). At 12 and 24 months after the baseline visit (y‐aa and ab‐ad, respectively), SRF on OCT was still absent (y and ab). No marked changes were seen on fundus photograph (z and ac) and FAF (aa and ad), comparing imaging at 24 months to the evaluation after eplerenone treatment.

The patients who were randomized to half‐dose PDT (see Figure 3) showed a significantly higher rate of complete SRF resolution at 3 months after treatment, compared to treatment with oral eplerenone (van Rijssen et al., 2022). In addition, after crossover treatment to half‐dose PDT, a significant percentage of cCSC patients who primarily received eplerenone exhibited SRF resolution, indicating the importance of half‐dose PDT in the treatment of cCSC (see Figure 4) (Feenstra, van Dijk, et al., 2023; Feenstra, van Dijk, Rijssen, Tsonaka, Diederen, Schlingemann, & Boon, 2022). Based on the results of the current study conducted at 24 months after primary treatment, a 3‐month delay before half‐dose PDT is performed in cCSC patients does not lead to significantly worse anatomical and functional long‐term outcomes. This is especially important when there is a shortage of verteporfin, which is required for performing PDT (Sirks et al., 2022, 2024). Similarly, there were no significant differences in the odds ratio for the presence of SRF between baseline visit and final visit in patients primarily randomized to eplerenone compared to primary randomization to half‐dose PDT. In this regard, it is important to note that 32 of the 40 patients in the eplerenone group received half‐dose PDT as crossover treatment, whereas only 8 of 40 patients in the primary half‐dose PDT required crossover treatment with eplerenone (Feenstra, van Dijk, et al., 2023; Feenstra, van Dijk, Rijssen, Tsonaka, Diederen, Schlingemann, & Boon, 2022).

This study showed that half‐dose PDT can significantly decrease SFCT in cCSC patients, in contrast to eplerenone, for at least 2 years after treatment. Choroidal abnormalities such as venous overload, increased choroidal thickness and choroidal hyperpermeability are characteristic for CSC, and the superiority of half‐dose PDT over other treatment modalities in treating cCSC may lie for a large part in its ability to positively affect these choroidal abnormalities (Cheung et al., 2024; Feenstra et al., 2021; Pauleikhoff et al., 2024a, 2024b; Spaide et al., 2022). Half‐dose (or half‐fluence) PDT has recently been advocated as preferred first‐line treatment for cCSC in the first evidence‐based treatment guideline for CSC by an international group of experts, and the current study lends further support to this recommendation (Feenstra et al., 2024). The increase in CRT observed in both treatment groups at final visit compared to baseline visit is an interesting finding. In contrast to our study, some studies have reported a significant reduction in CRT in cCSC patients treated with half‐dose PDT, which may mostly be due to the inadvertent inclusion of the SRF into the CRT measurements in these studies (Liu et al., 2014; Nicolo et al., 2012; Pichai et al., 2021; Zhao et al., 2015). In the current study, a specific manual CRT measurement was performed for each case, defining CRT as the distance between the internal limiting membrane and the inner border of the ellipsoid zone, as described by Van Rijssen et al, which—importantly—does not include the SRF (van Rijssen, Mohabati, et al., 2018; van Rijssen, van Dijk, et al., 2018). The specific reasons for the increase in CRT observed in our study are unknown and require further investigation. Possibly, retinal thinning occurs during the presence of SRF in the active phase of CSC, which could be normalized by PDT (van Rijssen, Mohabati, et al., 2018; van Rijssen, van Dijk, et al., 2018).

In terms of foveal sensitivity, the half‐dose PDT group demonstrated a significant increase of + 3.9 dB at evaluation visit 1, significantly surpassing the comparatively lower increase of + 1.7 dB observed in the eplerenone group at this initial evaluation visit (van Rijssen et al., 2022). Following crossover treatment to half‐dose PDT in the 88.1% of patients in primary eplerenone group who had persistent SRF at evaluation visit 1, a notable improvement in foveal sensitivity was observed, highlighting the superiority of half‐dose PDT over eplerenone in achieving an increase in foveal sensitivity (Feenstra, van Dijk, et al., 2023; Feenstra, van Dijk, Rijssen, Tsonaka, Diederen, Schlingemann, & Boon, 2022). At the time of the final visit, there was no significant difference in foveal sensitivity between the patients primarily randomized to half‐dose PDT and eplerenone nor were there any significant differences in BCVA increase at this final visit. This indicates that a delay of 3 months before implementing half‐dose PDT leads to similar results compared to immediate PDT. However, the effects of longer delays (e.g. 6–12 months) remain unknown and warrant further investigation. It is unknown if longer delays may lead to significant differences in structural and functional outcomes, although there are studies suggesting that persistent and/or recurrent SRF on OCT in cCSC are indeed associated with worse outcome (Mohabati, Schellevis, et al., 2018; Mohabati, van Dijk, et al., 2018). It is also unknown to what extent prolonged presence of SRF and disease activity can contribute to complications such as MNV and the development of non‐neovascular intraretinal fluid collections, also known as posterior cystoid retinal degeneration (Mohabati, Boon, & Yzer, 2020; Mohabati, Hoyng, et al., 2020; Mohabati, Schellevis, et al., 2020). Given the fact that PDT has such a pronounced beneficial effect on SRF resolution and functional improvement, it decreases the risk of SRF recurrence and has shown to be safe even when including the fovea, early treatment should be strongly considered if possible (Feenstra, Diederen, et al., 2023; Feenstra, van Dijk, et al., 2023; van Rijssen et al., 2021). A long‐term follow‐up study by Vasconcelos et al. showed that morphological an functional chorioretinal changes observed 5 years of more after full‐dose PDT were not correlated with the location of treatment, which also indicates that half‐dose PDT is safe long term (Vasconcelos et al., 2013).

During the course of the study, no noteworthy adverse events occurred. Importantly, none of the patients had an increase of foveal atrophy (Feenstra, Diederen, et al., 2023; Feenstra, van Dijk, et al., 2023). Some patients had pre‐existent mild atrophic changes in the macula, which are considered to be part of the natural disease course of cCSC (Mohabati, Schellevis, et al., 2018; Mohabati, van Dijk, et al., 2018; Mohabati, van Rijssen, et al., 2018). A total of eight patients had developed an MNV, including five in patients who were primarily randomized to eplerenone and three who were randomized to half‐dose PDT. All of these patients received crossover treatment. The development of those MNVs is most likely part of the disease course of CSC, with reported rates as high as 36% among cCSC patients prior to receiving treatment (Peiretti et al., 2015; Serra et al., 2022; Zhou et al., 2022). Notably, all MNV cases occurred in patients who required crossover treatment due to persistent SRF, suggesting that prolonged disease activity may be a risk factor.

Our study has several important limitations. First, the fact that not all included patients in the SPECTRA trial could have been analysed at the final visit (mainly due to loss to follow up) may restrict the generalizability of the findings and limits the statistical power of the analysis. In addition, the crossover treatment among a significant number of patients introduces a limitation that prevents the feasibility of conducting analyses solely focused on one specific treatment. Lastly, the current study included patients who had SRF on OCT or visual complaints for at least 6 weeks but for no longer than 18 months. However, exact information about the baseline duration of SRF at recruitment was not present. Considering these limitations, the results should be interpreted in this context; additional studies would be valuable to validate and expand upon the findings of this study.

In summary, the findings of this study provide compelling long‐term evidence for the safety and efficacy of half‐dose PDT in effectively resolving SRF and improving functional outcomes in cCSC patients. The results not only support the use of half‐dose PDT as a viable immediate treatment option in this disabling chorioretinal disease but also shed light on the benefits that can still be achieved after a delayed initiation, being especially of importance during a period in which there is a shortage of verteporfin. Nevertheless, early administration of PDT should be considered in cCSC to optimize the likelihood of SRF resolution, vision improvement and reduction of the risk of disease recurrence, as recommended in a recent evidence‐based treatment guideline (Feenstra et al., 2024).

## FUNDING INFORMATION

The author(s) have no proprietary or commercial interest in any materials discussed in this article.

## Supporting information


Figure S1.

Table S1.

Table S2.
