# Supplementary material for: Long‐term follow‐up of chronic central serous chorioretinopathy patients receiving oral eplerenone and half‐dose photodynamic therapy in the SPECTRA trial: SPECTRA trial report No. 4
Source: Acta Ophthalmol. 2026 Feb 11;104(5):e565–77. doi: 10.1111/aos.70106 (PMC13353556; doi:10.1111/aos.70106)
Supplement: Supplementary file 1 — Figure S1. [file AOS-104-e565-s001.docx]

Baseline visit

Eplerenone treatment (after laboratory tests)

Half-dose PDT (within 4 weeks after baseline visit)

3 months

3 months

Evaluation visit 1: no SRF on OCT

Evaluation visit 1: no SRF on OCT

Evaluation visit 1: SRF on OCT

Evaluation visit 1: SRF on OCT

Eplerenone treatment (after laboratory tests)

Half-dose PDT (within 4 weeks after evaluation visit 1)

9 months

3 months

3 months

9 months

Evaluation visit 2: no SRF on OCT

Evaluation visit 2: SRF on OCT

Evaluation visit 2: no SRF on OCT

Evaluation visit 2: SRF on OCT

6 months

6 months

Follow-up/ treatment at the discretion of the treating ophthalmologist

Final visit (24 months after baseline visit)

Evaluation visit 3 (12 months after baseline visit)
