# Supplementary material for: Long‐term follow‐up of chronic central serous chorioretinopathy patients receiving oral eplerenone and half‐dose photodynamic therapy in the SPECTRA trial: SPECTRA trial report No. 4
Source: Acta Ophthalmol. 2026 Feb 11;104(5):e565–77. doi: 10.1111/aos.70106 (PMC13353556; doi:10.1111/aos.70106)
Supplement: Supplementary file 2 — Table S1. [file AOS-104-e565-s003.docx]

|  | Half-dose photodynamic therapy group (n=53) | Eplerenone group (n=54) | Total (n=107) | *p*-value |
| --- | --- | --- | --- | --- |
| Mean age (years) | 44.5 ± 10.0 | 47.5 ± 9.6 | 46.0 ± 9.9 | 0.118 |
| Male gender | 51 (96%) | 49 (91%) | 100 (94%) | 0.251 |
| Mean BCVA (ETDRS letters) | 78.0 ± 13.1 | 80.5 ± 7.9 | 79·3 ± 10·8 | 0.236 |
| Mean retinal sensitivity on microperimetry (dB) | 22.7 ± 4.3 | 22.5 ± 4.1 | 22.6 ± 4.2 | 0.800 |
| Mean foveal sensitivity on microperimetry (dB) | 20.1 ± 4.6 | 20.0 ± 4.7 | 20.1 ± 4.6 | 0.762 |
| Mean NEI-VFQ-25 composite score (points) | 81.7 ± 11.3 | 79.5 ± 13.1 | 80.5 ± 12.2 | 0.350 |
| Subfoveal choroidal thickness (µm) | 363.9 ± 77.1 | 401.4 ± 128.8 | 384.4 ± 108.3 | 0.128 |
| Central retinal thickness (µm) | 108.0 ± 20.0 | 104.0 ± 19.0 | 105.8 ± 19.5 | 0.333 |

BCVA = best-corrected visual acuity; dB = decibel; ETDRS = Early Treatment of Diabetic Retinopathy Study; NEI VFQ-25 = National Eye Institute Visual Functioning Questionnaire 25-item version; SPECTRA = Half-Dose Photodynamic Therapy Versus Eplerenone in Chronic Central Serous Chorioretinopathy.

Data are mean ± SD or number (%).
