# Supplementary material for: Long‐term follow‐up of chronic central serous chorioretinopathy patients receiving oral eplerenone and half‐dose photodynamic therapy in the SPECTRA trial: SPECTRA trial report No. 4
Source: Acta Ophthalmol. 2026 Feb 11;104(5):e565–77. doi: 10.1111/aos.70106 (PMC13353556; doi:10.1111/aos.70106)
Supplement: Supplementary file 3 — Table S2. [file AOS-104-e565-s002.docx]

|  | Primarily randomized to half-dose photodynamic therapy (n=53) | | Primarily randomized to eplerenone (n=54) | |
| --- | --- | --- | --- | --- |
|  | Number of patients (%) | Number of events | Number of patients (%) | Number of events |
| All adverse events | 16 (30.2%) | 22 | 30 (55.6%) | 41 |
| Serious adverse events | 0 | 0 | 0 | 0 |
| Number of patients reporting the following symptoms throughout the trial | | | | |
| Stomach complaints after taking medication (not eplerenone) | 1 (not treatment-related) | |  | |
| Neovascularization in study eye* | 3 (not treatment-related) | | 5 (not treatment-related) | |
| Dizziness |  | | 3 (possibly treatment-related) | |
| Headache |  | | 3 (possibly treatment-related) | |
| Skin rash | 1 (not treatment-related) | | 2 (not treatment-related) | |
| Paresthesia hand or leg | 1 (possibly treatment-related) | | 2 (possibly treatment-related) | |
| Cataract extraction | 1 (not treatment-related) | | 1 (not treatment-related) | |
| Fatigue, poor quality of sleep | 1 (not treatment-related) | | 1 (not treatment-related) | |
| Stomach complaints |  | | 2 (possibly treatment-related) | |
| Vasovagal reaction during fluorescein angiography |  | | 2 (possibly treatment-related) | |
| Background diabetic retinopathy | 1 (not treatment-related) | |  | |
| Burn-out |  | | 1 (not treatment-related) | |
| Cataract (not treated) |  | | 1 (not treatment-related) | |
| Conjunctivitis in both eyes |  | | 1 (not treatment-related) | |
| Cornea erosion | 1 (not treatment-related) | |  | |
| Cystitis |  | | 1 (not treatment-related) | |
| Dental pain | 1 (not treatment-related) | |  | |
| Diarrhea |  | | 1 (possibly treatment-related) | |
| Discovery of benign kidney cysts |  | | 1 (not treatment-related) | |
| Eczema | 1 (not treatment-related) | | 1 (not treatment-related) | |
| Epistaxis | 1 (not treatment-related) | |  | |
| Folliculitis | 1 (not treatment-related) | |  | |
| General malaise |  | | 1 (possibly treatment-related) | |
| Head injury | 1 (not treatment-related) | |  | |
| Heart palpitations |  | | 1 (possibly treatment-related) | |
| High glucose levels during routine check | 1 (not treatment-related) | |  | |
| Itchy eye lid |  | | 1 (possibly treatment-related) | |
| Lower back pain | 1 (not treatment-related) | |  | |
| Nausea after taking medication (varenicline) |  | | 1 (not treatment-related) | |
| Nausea |  | | 1 (possibly treatment-related) | |
| Nipple tenderness |  | | 1 (possibly treatment-related) | |
| Otitis |  | | 1 (not treatment-related) | |
| Planned abdominal surgery |  | | 1 (not treatment-related) | |
| Removal of benign warts |  | | 1 (not treatment-related) | |
| Rib contusion | 1 (not treatment-related) | |  | |
| Rhinitis |  | | 1 (not treatment-related) | |
| Sprained ankle |  | | 1 (not treatment-related) | |
| Swollen finger |  | | 1 (not treatment-related) | |
| Tonic-clonic seizure | 1 (not treatment-related) | |  | |
| Venous insufficiency with edema in legs | 1 (not treatment-related) | |  | |
| Tooth extraction | 1 (not treatment-related) | |  | |
| Vertigo | 1 (not treatment-related) | |  | |
| Visual complaints in both eyes |  | | 1 (possibly treatment-related) | |
